# Pervasive Discrimination is Associated With Internal Health Locus of Control in the MIDUS Study

**DOI:** 10.1093/geroni/igaf122.3639

**Published:** 2025-12-31

**Authors:** Carolina Hamatsu, David Almeida, Alexis Santos

**Affiliations:** Pennsylvania State University, University Park, Pennsylvania, United States; Pennsylvania State University, University Park, Pennsylvania, United States; Pennsylvania State University, University Park, Pennsylvania, United States

## Abstract

**Objectives:**

Drawing on Learned Helplessness Theory, the current study explores the association between Internal Health Locus of Control (I-HLOC) and Pervasive Discrimination (PD). We hypothesize that higher levels of PD are associated with a lower I-HLOC, potentially reflecting the impact of cognitive and behavioral processes shaped by repeated experiences of discrimination.

**Method:**

I-HLOC and a PD composite score were calculated using data from the second wave of the Midlife in the United States study (MIDUS II), a nationally representative sample of adults in the United States (n = 2,406). Linear regression models were conducted to study the association between PD and I-HLOC and its dimensions (i.e., everyday, lifetime, and workplace discrimination), while controlling for age, sex, race, marital status, income level, employment status, and educational attainment.

**Results:**

We found that individuals in the top tertile on at least one dimension of discrimination reported lower I-HLOC. Separate models for each dimension revealed significant associations for the top tertile of everyday discrimination and workplace discrimination with lower levels of I-HLOC. No association was found for lifetime discrimination.

**Discussion:**

Our findings suggest a potential link between I-HLOC and PD. The main contributors are having experienced everyday and workplace discrimination, lending support to our hypothesis. These findings offer a promising explanation for how intra-individual cognitive and behavioral processes may contribute to health disparities by shaping personal beliefs about the level of control people perceive they have when living in the context of repeated or chronic experiences of unfair treatment in the United States.

